# Impact of training in clinical and microscopy diagnosis of childhood malaria on antimalarial drug prescription and health outcome at primary health care level in Tanzania: A randomized controlled trial

**DOI:** 10.1186/1475-2875-7-199

**Published:** 2008-10-02

**Authors:** Billy Ngasala, Marycelina Mubi, Marian Warsame, Max G Petzold, Amos Y Massele, Lars L Gustafsson, Goran Tomson, Zul Premji, Anders Bjorkman

**Affiliations:** 1 Infectious Diseases Unit, Department of Medicine, Karolinska University Hospital, Karolinska Institutet, Stockholm, Sweden; 2 Department of Parasitology, Muhimbili University of Health and Allied Sciences, Dar es Salaam, Tanzania; 3 Division of International Health, Karolinska Institutet, Stockholm, Sweden; 4 The Nordic school of Public Health, Gothenburg, Sweden; 5 Department of Pharmacology, Muhimbili University College of Health Sciences, Dar es Salaam, Tanzania; 6 Division of Clinical Pharmacology, Department of Laboratory Medicine, Karolinska Institute, Stockholm, Sweden; 7 Currently at Global Malaria Programme, World Health Organization, Geneva, Switzerland

## Abstract

**Background:**

Prescribing antimalarial medicines based on parasite confirmed diagnosis of malaria is critical to rational drug use and optimal outcome of febrile illness. The impact of microscopy-based versus clinical-based diagnosis of childhood malaria was assessed at primary health care (PHC) facilities using a cluster randomized controlled training intervention trial.

**Methods:**

Sixteen PHC facilities in rural Tanzania were randomly allocated to training of health staff in clinical algorithm plus microscopy (Arm-I, n = 5) or clinical algorithm only (Arm-II, n = 5) or no training (Arm-III, n = 6). Febrile under-five children presenting at these facilities were assessed, treated and scheduled for follow up visit after 7 days. Blood smears on day 0 were only done in Arm-I but on Day 7 in all arms. Primary outcome was antimalarial drug prescription. Other outcomes included antibiotic prescription and health outcome. Multilevel regression models were applied with PHC as level of clustering to compare outcomes in the three study arms.

**Results:**

A total of 973, 1,058 and 1,100 children were enrolled in arms I, II and III, respectively, during the study period. Antimalarial prescriptions were significantly reduced in Arm-I (61.3%) compared to Arms-II (95.3%) and III (99.5%) (both P < 0.001), whereas antibiotic prescriptions did not vary significantly between the arms (49.9%, 54.8% and 34.2%, respectively). In Arm-I, 99.1% of children with positive blood smear readings received antimalarial prescriptions and so did 11.3% of children with negative readings. Those with positive readings were less likely to be prescribed antibiotics than those with negative (relative risk = 0.66, 95% confidence interval: 0.55, 0.72). On day 7 follow-up, more children reported symptoms in Arm-I compared to Arm-III, but fewer children had malaria parasitaemia (p = 0.049). The overall sensitivity of microscopy reading at PHC compared to reference level was 74.5% and the specificity was 59.0% but both varied widely between PHCs.

**Conclusion:**

Microscopy based diagnosis of malaria at PHC facilities reduces prescription of antimalarial drugs, and appears to improve appropriate management of non-malaria fevers, but major variation in accuracy of the microscopy readings was found. Lack of qualified laboratory technicians at PHC facilities and the relatively short training period may have contributed to the shortcomings.

**Trial registration:**

This study is registered at Clinicaltrials.gov with the identifier NCT00687895.

## Background

The annual burden of *Plasmodium falciparum *in sub-Saharan Africa is estimated to be 365 million clinical episodes resulting in over one million fatal cases, mostly in children underfive years of age [1]. In Tanzania malaria continues to be the leading cause of morbidity and mortality accounting for about 30% and 15% of all hospital admissions and deaths, respectively [2].

Prompt and effective case management is critical for the successful implementation of malaria control strategy [3]. Although over 80% of Tanzanians live within 5 kms of a health facility providing malaria treatment, malaria case management is often inadequate. In many primary health care (PHC) facilities in Tanzania the quality of malaria diagnosis and treatment suffers from inadequate clinical examination, lack of diagnostic equipment, such as microscopes, and non compliance with standard malaria treatment guidelines [4-6]. Inappropriate drugs and inadequate treatment will cause higher morbidity and mortality from malaria [7], may also enhance the development of drug resistance [8] and prevent optimal management of fever illness other than malaria.

Most of malaria endemic African countries have adopted the use of highly effective but expensive artemisinin-based combination therapy (ACT). Restricted and better directed treatment is increasingly advocated to prevent drug resistance and reduce the costs of these drug combinations [9,10]. Efforts have been made to improve the management of illnesses in children, including malaria, through the Integrated Management of Childhood Illnesses (IMCI) strategy. However, the IMCI algorithm has shown poor specificity in diagnosing malaria and causes over-treatment with antimalarial drugs [11,12]. This was less of a concern in the era of inexpensive chloroquine, compared to the present use of expensive antimalarial drugs like ACT. Improved diagnostic capacity potentially includes the use of microscopy which still remains the gold standard for laboratory diagnosis of malaria [5,6]. New diagnostic methods including malaria rapid tests (RDTs) also need to be considered, but have limitations, such as batch to batch quality variation, no parasite density determination, persistent positivity after treatment, accuracy and cost [13,14].

The establishment and maintenance of reliable and efficient malaria microscopy at PHC level requires organized health system infrastructure, with functioning microscopes, well trained health workers, regular provision of reagents, supervision and quality control [15,16]. This intervention study aimed to assess the impact of training in microscopy and clinical diagnosis on antimalarial drug prescriptions and health outcomes in childhood malaria at PHC level.

## Methods

### Study area

The study was conducted in two neighbouring districts, Kibaha and Bagamoyo, situated in the Coast region, north of Dar es Salaam Tanzania, and with estimated populations of about 103,000 and 240,000, respectively. They have similar geographical characteristics. Malaria transmission is holoendemic, with some accentuation during the rainy seasons in May-July and December to January. *Plasmodium falciparum *is the dominant parasite species. In Kibaha the government health facilities consist of one hospital and 19 Primary Health Care (PHC) facilities (two health centres and 17 dispensaries) and Bagamoyo has one hospital and 41 PHC facilities (four health centres and 37 dispensaries). Most PHC facilities are in the rural areas where the majority of the population live. The health staff includes assistant medical officers (AMO), clinical officers (CO), assistant clinical officers (ACO), mother and child health aides (MCHA), trained nurses, nurse auxiliaries and laboratory assistants. They are supervised by the district medical officer (DMO) and other senior health officers from the district hospital.

PHC facilities receive medicines including antimalarial drugs monthly through the essential drug program-Kit system from the Medical Store Department (MSD), a semi-autonomous institution located in part under the Ministry of Health. Microscopes are available in health centers and some dispensaries, but their use is limited because of lack of qualified/skilled laboratory technicians. At the time of study, sulphadoxine/pyrimethamine (SP) and amodiaquine (AQ) were the first- and second-line treatments for uncomplicated malaria and quinine (QN) the drug of choice for severe malaria.

### Study design and allocation of health facilities

**Figure 1:**
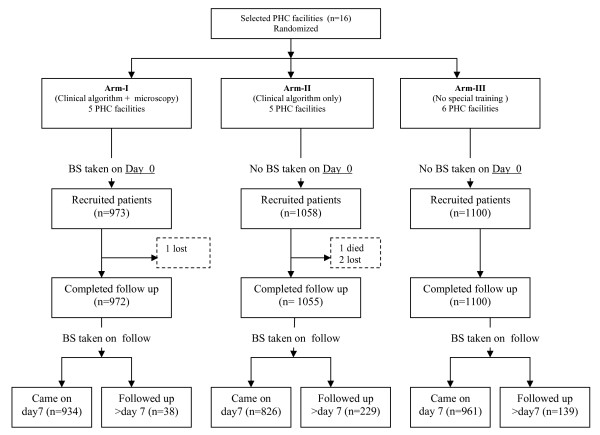
**Study design and number of patients recruited to the study and follow up visits by type of intervention.** PHC = primary health care. BS = blood smear.

The study was designed as a cluster-randomized controlled intervention evaluating two intervention packages (Figure 1). PHC facilities were eligible for the study if they were rural government owned; accessible by road during rainy season; and within three hours by car from Muhimbili University College of Health Sciences (MUCHS), Dar es Salaam. Out of 62 PHC facilities in the two districts, 31 qualified and visited by study team. The study objectives and procedures were explained to the health workers and a total of 16 PHC facilities consented and signed their commitment to participate: three health centres (one in Kibaha and two in Bagamoyo district) and 13 dispensaries (five in Kibaha and nine in Bagamoyo district). The PHC facilities were stratified into those with previous exposure to IMCI training and those without and randomly allocated either to training in clinical algorithm plus microscopy (Arm-I, n = 5), or clinical algorithm only (Arm-II, n = 5), or to no training (Arm-III, n = 6). The remaining 15 PHC facilities did not participate because they were engaged in other ongoing research activities.

### Patients

Children attending the study PHC facilities during daytime were enrolled if they fulfilled the following criteria: a) under five years of age, b) fever (axillary temperature ≥ 37.5°C) and/or history of fever in last two days, c) able to return to the facility on day 7 after treatment or any other day if symptoms were to worsen or recur and d) the mother/guardian consented to participate. Patients with severe illness requiring inpatient care according to the IMCI guidelines were admitted or referred to the health centres or the district hospitals. Both consultation and treatments are provided free for under-five children.

### Intervention packages

#### Clinical diagnosis of malaria

Based on national malaria treatment and the IMCI guidelines, a clinical training package was developed aiming at improving the knowledge and the skills of the health workers to clinically diagnose and treat malaria. The content of the package included (a) description of signs and symptoms of malaria disease; (b) history taking relevant to malaria and physical examination; (c) identification of danger signs and severe illness for referral; (d) appropriate treatment; (e) counselling patients on the use of drugs.

#### Microscopy diagnosis of malaria

Based on the World Health Organization (WHO) basic malaria microscopy training manual [17], a training package on malaria microscopy was developed on how to (a) make thick blood smears from febrile patients and stain with Giemsa; (b) identify and count malaria parasites; (c) maintain the microscope and store blood slides. Training in haemoglobin (Hb) estimation using the Tallquist test was also included in the package.

#### Training modules

The training for both packages included presentations of theoretical information, brain storming and group discussion. Clinical demonstrations were provided by the research team illustrating how to take history, physical examination, diagnosis, prescribing and counselling. There were also clinical practice sessions with the health care workers assessing patients under supervision with individual feedback and practical sessions on malaria microscopy which included blood smear preparation and reading, parasite counting and artefact identification. A pilot training programme conducted in rural Dar es Salaam PHC facilities demonstrated its feasibility and feedback from the participants was used to improve the final training programme.

Health workers in Arm-I and II facilities received five-day group training on management of malaria on clinical basis and the health workers in Arm-I received an additional five-day group training in malaria microscopy. No training was provided to those in Arm-III. In all three arms, health workers were trained on how to fill the case record forms and how to prepare malaria blood smears. All health facility staff involved in the care of under-fives were included in the training i.e. AMOs, COs, ACOs, MCHAs, nurses, nurse assistants and laboratory assistants/attendants. In Arm-I, microscopy was performed by assistant laboratory technicians with one year training in two PHC facilities (Chalinze and Mwanabwito) and by the COs in three facilities (Kikongo, Mbwewe and Ubena estate).

The training was held at each health facility during working hours; practical training was conducted in morning sessions and the theoretical part in afternoons. The training was facilitated by two PhD research students (Ngasala B and Mubi M) assisted by a senior laboratory technician from MUCHS and supervised once a week by senior members of the team. The project provided all study health facilities with all supplies needed for making blood smears. In addition each health facility in Arm-I was provided with one light microscopy and malaria microscopy guidelines.

### Data collection

Children presenting at the study facilities with fever were enrolled in the study and information on age, sex, duration of fever, other presenting symptoms and body temperature was recorded. Assessment of body temperatures (axillary) were performed by using mercury thermometers. In Arm-I, a thick blood smear for malaria was collected from each patient and examined by the health worker (first reading) and haemoglobin levels were estimated using the Tallquist test. The collected blood smears were forwarded weekly to MUCHS for second reading by an experienced laboratory technician for malaria microscopy. In Arm-II and Arm-III, blood smears for malaria and Tallquist test were not done on day 0. Diagnosis and prescribed drugs (antimalarial drugs, antibiotics, antipyretic and other drugs as per national treatment and IMCI guidelines) were recorded at the end of the consultations.

In all three arms, patients were scheduled for day 7 follow-up visits and encouraged to report at any time prior to day 7 if necessary. The health workers assessed them for new or persistent symptoms, any concomitant medication taken and any adverse event perceived. In addition, thick blood smears were taken during the follow-up visits and examined by the second reader at MUCHS. Patients who failed to attend the health facility for day 7 follow up were visited at home to assess their health status and to identify possible reasons for not attending scheduled follow up.

At the end of the study special interviews were conducted with health workers in Arm-I to assess whether and under what circumstances they prescribed antimalarial treatment to patients with negative blood smear results.

### Laboratory examinations

Thick blood smears were stained with 5% Giemsa for 20 minutes. Microscopy reading at PHC level included species identification and parasite count. The parasite density was estimated by counting the number of asexual parasites per 200 white blood cells and calculating parasites per μL, assuming a white cell count of 8,000 cells per μL. A smear was declared negative if no asexual parasites were seen after examining 100 high power fields. Every week an experienced microscopist at MUCHS re-examined all blood smears without knowledge of the first reading. The result of the second reading was compared with the first reading and if discordant results (positive vs. negative) or even two fold difference in parasite density, a third decisive microscopy reading, again blinded was to be done.

Using a Tallquist paper scale method, a drop of blood absorbed on a test strip of filter paper was matched against the scale standards by moving it up and down, to determine the equivalent Hb value. The Tallquist paper scale has colours which range from yellow to brown in a scale of 10%–100% which corresponds to Hb range from 1.58 to 15.8 g/dL.

### Outcome measures

The primary outcome was the proportion of study children receiving prescriptions of antimalarial drugs in the respective arms. Secondary outcomes included prescriptions of antibiotics, cost of drugs and health outcome of the patients, i.e. the proportion of patients re-visiting the health facility due to clinical complaints on days 1 to 6 and the proportion reporting symptoms on day 7 follow-up. Full health improvement was determined by the proportion of the study patients in each arm alive and without complaints of fever or any other symptoms and without parasitaemia on day 7. The cost of drugs was calculated using price list from Medical Store Department. Drug costs for each patient were calculated based on the specific weight or age related drug regimen costs.

### Sample size and statistical analysis

The prescription rate of antimalarial drugs was assumed to be 90% in the control arm and the intervention package in Arm-I was assumed to reduce this to 70%. The minimum sample size for independent observations to give 90% power at a significance level of 5% was found to be 140 patients per comparative arm, when adjusted for multiple testing (Bonferroni). To account for secondary outcomes, potential clustering at PHC level and a loss to follow up, we decided to recruit 200 patients per facility giving approximately 1,000 patients per arm. Comparisons of the differences in outcomes for the study arms multilevel regression models were applied with PHC as level of clustering (XTMIXED command in STATA version 10). In the result section 95% confidence intervals (CI) are given and stated significances are on the 5% level. Descriptive results within treatment arms were not adjusted for clustering due to limited number of PHCs per treatment arm.

### Ethical considerations

Approval of the study was granted by the National Institute for Medical Research Tanzania and the Karolinska Institutet (D-nr 03-712) ethical committees. Permission to carry out the study was obtained from the district health team, wards authorities and village leaders and PHC facility workers. Informed written consent was sought from parents/guardians of the under-five children.

## Results

### Baseline characteristics

A total of 3,131 children with fever were enrolled between July 2003 and March 2004 (Figure 1). The baseline characteristics of the enrolled children in the three arms are summarized in Table 1. Age and sex distributions were similar. Almost all patients in the three study arms had a temperature measured and recorded during their visit on day 0 (i.e. 99.6%, 96.1% and 96.8% in arms I, II and III, respectively). Proportions of children with recorded temperature ≥ 37.5°C were 50.4% in Arm-II compared to over 70% in the other two arms (p < 0.001). Msoga and Lugoba health facilities mainly contributed to the low rate of fever recorded in Arm-II. The distributions of medications reported taken by patients two weeks prior to visit at health facilities were comparable across the study arms. Prior self-treatment with antipyretics mostly paracetamol was common (around 50%) while only 1% and 2% of children reported medication with antimalarial drugs and antibiotics, respectively. Less than 1% (range 0.5%–0.6%) of patients enrolled in each arm were referred on day 0 (n = 6, Arm-II, n = 5, Arm-III, n = 6). In Arm-I, 361/934 (38.7%) children tested positive for malaria parasitaemia according to the blood smear readings at MUCHS, the geometric mean (range) was 5,247 (120–200,000) parasites/μL. In Arm-I, using Tallquist test, 665 children (71.7%) were considered anaemic (Hb range 50%–70%), and 172 (17.7%) severely anaemic (Hb < 50%).

**Table 1:** Baseline characteristics of the study children enrolled in the three intervention arms

Characteristics	Clinical algorithm + microscopy (Arm-I)	Clinical algorithm (Arm-II)	No special training (Arm-III)
Total children enrolled	973	1058	1100
Mean age in months (SD)	22.5 (14.9)	20.0 (14.8)	20.4 (14.3)
Males	472 (48.5)	555 (52.5)	563 (50.8)
Prior treatment taken*			
Paracetamol	520 (53.4)	522 (49.3)	442 (40.1)
Sulfadoxine-pyrimethamine	10 (1.0)	9 (0.9)	8 (0.7)
Amodiaquine	7 (0.7)	12 (1.1)	5 (0.5)
Antibiotics	11 (1.1)	22 (2.1)	6 (0.6)

Values are numbers (percentages) unless stated otherwise

*14 days prior to enrolment

### Prescription of drugs

Table 2 summarizes drug prescription patterns in the three study arms. The frequency of antimalarial prescriptions was significantly lower in Arm-I (61.0%, CI: 51.6%, 70.3%) compared to Arm-II (95.2%, CI: 85.7%, 100%) and control Arm-III (99.4%, CI: 90.9%, 100%) (both *p *< 0.001). SP, officially first-line antimalarial drug, was prescribed to most malaria patients (range 73.8%–100%) in 14 study health facilities and AQ, officially second-line antimalarial drug, was prescribed to most patients (48% and 79%) in the two remaining health facilities (one in Arm-I and another in Arm-III). Overall, in accordance with Tanzania national treatment guidelines 76.7% of antimalarial drugs were correctly (dosage and duration of treatment) prescribed; 9.8% were under-dosed; 10.2% were over-dosed; and in 3.3%, the treatment was correct but the prescription indicated insufficient duration. There was no significant difference in the proportion of antimalarial drugs correctly prescribed in the three arms.

**Table 2:** Medicines prescribed to febrile children attending the study PHC facilities in the three study arms.

Parameters	Clinical algorithm + microscopy (Arm-I)	Clinical algorithm (Arm-II)	No special Training (Arm-III)
Total number of patients	973	1058	1100
Patients with prescriptions	944 (97.0)	1057 (99.9)	1097 (99.7)
Antimalarial drugs	576 (61.0) (CI: 51.6, 70.3)	1008 (95.2) (CI: 85.7, 100)	1091 (99.4) (CI: 90.9, 100)
Antibiotics	434 (45.9) (CI: 28.1, 63.7)	593 (54.8) (CI: 37.1, 72.6)	375 (34.2) (CI: 17.9,50.5)
Antipyretics	903 (95.7)	1027 (97.2)	1041(94.9)
Number of drugs prescribed	2306	2961	2718
Number of drugs per prescription	2.5	3.0	2.5
Average cost per antimalarial treatment	0.083 US$	0.072 US$	0.062 US$
Average cost of antimalarial drug/patient	0.050 US$	0.070 US$	0.062 US$
Average cost of all drugs/patient	0.200 US$	0.250 US$	0.170 US$

Values are numbers (percentages).

CI = (95% confidence interval).

1 US$ = 1000 Tanzanian shillings.

In all antibiotics were prescribed to 44.8% of all patients but there was no significant difference in the proportion of antibiotic prescriptions in the three arms (Table 2). The antibiotics prescribed included tablets, capsules and syrups (penicillin, ampicillin, amoxycillin, ampicloxacillin, erythromycin and co-trimoxazole), injectables (gentamycin, procaine and benzyl penicillin) and topical preparation (tetracycline eye ointment). The most common antibiotics prescribed in all three arms were co-trimoxazole and injectable procaine penicillin and benzyl penicillin. Almost all patients in the three arms were prescribed antipyretics (Table 2).

The average number of drugs per prescription was higher in Arm-II compared to Arm-III (p = 0.003); other differences were not statistically significant. The average cost of all drugs in Arm-III was 0.170 US$. This increased by 47% in Arm-II (with clinical algorithm training) and by 18% in Arm-I (with microscopy) (Table 2).

### PHC versus MUCHS malaria microscopy

A total of 934 blood smears in Arm-I were re-examined at the reference laboratory at MUCHS by the experienced microscopist (Table 3). The first readings by the PHC workers and and the second readings by MUCHS microscopist agreed on a total of 607 (65%) blood smears, 269 positive smears (true positives) and 338 negative smears (true negatives). All third microscopy readings for discordant results agreed with second reading results. Overall sensitivity was 74.5% (CI: 69.8%, 78.7%) and specificity was 59.0% (CI: 54.9%, 62.9%). A positive predictive value of 53.4% (CI: 49.0%, 58.0%) and negative predictive value of 78.6% (CI: 74.0%, 82.0%) were observed. The accuracy of microscopy varied widely between the study PHC facilities, e.g. the negative predictive values were highest in Chalinze and Mwanabwito health facilities whereas the positive predictive values were highest in Kikongo and Mbwewe health facilities (Table 3). However, sensitivity of blood smear microscopy at PHC level increased with increasing parasite density (Table 4).

**Table 3:** Predictive values of blood smear (BS) results at Primary Health Care (PHC) facilities in the clinical algorithm + microscopy intervention arm

PHC Facilities	BS positive rate (PHC microscopy reading) n/N (%)	BS positive rate (MUCHS reading) n/N (%)	Positive predictive Value %	Negative predictive Value %
Chalinze	111/200 (55.5)	59/200 (29.5)	48.6	94.4
Ubena	140/180 (77.8)	83/176 (47.2)	51.1	66.7
Mbwewe	86/197 (43.7)	88/191 (46.1)	68.7	71.3
Kikongo	44/197 (22.3)	68/194 (35.1)	70.5	75.3
Mwanabwito	150/199 (75.4)	63/176 (36.4)	44.2	86.4

Total	531/973 (54.6) (CI: 51.4, 57.7)	361/934 (38.7)* (CI:35.6, 41.8)	53.4 (SD 12.1) (CI: 49.0, 58.0)	78.6 (SD 11.4) (CI: 74.0, 82.0)

CI = (95% confidence interval).

*39 Blood smear results missing/not done at reference laboratory by experienced microscopist

**Table 4:** Blood smear microscopy at Primary Health Care (PHC) facilities: sensitivity by parasite density in the clinical algorithm + microscopy intervention arm

Parasite density levels per μl blood	Number of patients (N = 361)	Sensitivity (%)
< 1000	63	49.2 (CI: 41.3, 57.1)
1,000 – 4,999	119	66.4 (CI: 57.9, 74.9)
5,000 – 10,0000	153	87.6 (CI: 82.4, 92.8)
> 100,000	26	96.2 (CI: 93.1, 99.2)

Total	361*	74.5 (CI: 67.6, 81.4)

CI = (95% confidence interval).

*934 blood smears were reviewed at reference level, 361 positive blood smears

In Arm-I and based on PHC facilities microscopy results, antimalarial drugs were prescribed to nearly all patients (> 99%, range 97.7%–100%) with positive blood smears but also to 50/442 (11.3%) with negative blood smears (Table 5). Patients with negative blood smears at Kikongo and Ubena health facilities were more likely to be prescribed antimalarial drugs (20% and 35%, respectively) whereas no or very few negative smear patients were treated at Mwanabwito, Chalinze and Mbwewe health facilities (0%, 1.1% and 3.6% respectively). According to the health workers at Kikongo and Ubena health facilities, patients with negative smears who received antimalarial drugs were mainly attended by nurse auxillaries/assistants when COs were away. All clinical staff stated they sometimes prescribe antimalarial drugs to patients despite negative microscopy results, particularly if a patient has high fever and no other identified cause of fever.

**Table 5:** Prescribed antimalarial drugs to febrile children in clinical algorithm + microscopy intervention arm in relation to blood smear (BS) microscopy results

	BS results at PHC (N = 973)		BS results at MUCHS (N = 934)*	
	
	BS positive	BS negative	BS positive	BS negative
Antimalarial prescribed	No (%)	No (%)	No (%)	No (%)
Yes	526 (99.1)	50 (11.3)	285 (78.9)	264 (46.1)
No	3 (0.5)	365 (82.6)	68 (18.8)	289 (50.4)
No record	2 (0.4)	27 (6.1)	8 (2.2)	20 (3.5)

Total	531	442	361	573

PHC = Primary health care – laboratory attendant

MUCHS = Muhimbili University College of Health Sciences- experienced microscopist

* 39 BS results missing/not done at reference laboratory

Antibiotics were prescribed to 193/531 (36.3%) patients in Arm-I with positive blood smears compared to 241/442 (54.5%) patients with negative smears, indicating that malaria patients were less likely to receive antibiotics compared to patients without parasitaemia (relative risk = 0.66, CI: 0.55, 0.72). Of the 442 patients with negative blood smears, 31% received neither antibiotic nor antimalarial drug i.e. only antipyretics and 4% had no record of any drug prescription. The influence of clinical malaria diagnosis on the antibiotic prescriptions could not be compared meaningfully in Arms II and III because almost all patients were considered as malaria and prescribed antimalarial drugs. Based on the readings of day 0 blood smears from Arm-I by the experienced microscopist at MUCHS, antimalarial drugs were prescribed to about 80% of the patients with parasitaemia and 46% without parasitaemia (Table 5).

### Health outcomes

A total of 74 patients re-visited the health facilities between days 1 to 6 with clinical complaints without significant difference between the arms (Table 6). Between day 1 and 7, 19 patients were referred to the district hospitals due to severe conditions which included severe malaria, severe pneumonia and severe anaemia. The frequency of referral was highest in Arm-II (P < 0.022). Of the referred patients, six patients re-attended between day 1 to 6 and 13 patients on day 7. Out of the 19 patients, 14 had received antimalarial treatment on day 0. One death was reported in Arm-II; the child had developed signs of severe malaria, four days after being prescribed SP and was referred but died in the district hospital.

**Table 6:** Health outcomes of the management of febrile childhood illness in the respective study Arms

Patient outcome	Clinical algorithm + microscopy (Arm-I) n/N (%)	Clinical algorithm (Arm-II) n/N (%)	No special training (Arm-III) n/N (%)
Re-attending on Day 1–6 with complaints	17/973 (1.7)	17/1058 (1.6)	40/1100 (3.6)
Re-attending on Day 1–6 with parasitaemia	8/17 (47.1)	10/17 (58.8)	24/40 (60)
Routine visit on Day 7 with complaints	94/934 (10.1)	47/826 (5.7)	41/961 (4.3)
Routine visit Day 7 with complaints and parasitaemia	19/94 (20)	7/47 (14.9)	10/41 (24.4)
Routine visit Day 7 with parasitaemia*	87/857 (10.2)	97/786 (12.3)	141/821 (17.2)
Referred Day 1–7	3/973 (0.3)	13/1058 (1.2)	3/1100 (0.3)
Death Day 1–7	0	1	0

*Blood smears missing from 77, 40 and 140 children in Arm-I, Arm-II and Arm-III respectively.

In Arm-I, 76 patients with positive blood smears according to the experienced microscopist did not receive antimalarial drugs on day 0 (Table 5). These patients were actively traced within 7 days of enrolment. One patient returned to the health facility on day 1 of the study with symptoms of severe malaria and anaemia, and was referred to the district hospital. Thirteen patients returned on day 2 of study with fever and parasitaemia and were successfully treated for uncomplicated malaria. In the remaining 62 patients, the fever resolved but parasitaemia persisted until they eventually received antimalarial treatment.

A total of 2,721 patients completed the follow up visit for the health outcome assessment at day 7 (± 1 day). In addition, 406 patients who did not turn up on day 7 follow up were visited in their homes on day 9 to 10. According to the post-interventions follow up at home; over 97% were reported to have recovered by day 7. The reasons provided by parents/guardians for not attending regular day 7 follow up included long distance to the facilities, travel or moved away and a few had no reasons.

About 10% of patients in Arm-I and 5% of patients in Arms-II and III had complaints of mainly fever, vomiting, diarrhoea or cough, but there was no statistically significant difference between the arms (Table 6). The frequency of patients with positive blood smears was, however highest in the control Arm-III (17.2%) compared to the intervention arms I (10.2%) and II (12.3%) (P = 0.049 and P = 0.21 respectively). Out of 325 patients with positive smears on day 7 from all three arms, 276 (84.9%) were positive despite having been prescribed antimalarial drugs on day 0. Types of antimalarial drugs taken by these patients on day 0 included; SP 78.6%, AQ 18.5% and QN 2.9%. In Arm-I, of the 466 patients with positive smear results, treated with antimalarial drugs on day 0 and re-examined on day 7; 424 (91%) became parasite negative ("cured").

When defining full health improvement as absence of fever or any symptoms or parasitaemia, the general recovery rates on day 7 were 695/934 (74.4%) patients in Arm-I, 649/826 (78.6%) in Arm-II and 650/961 (67.6%) in Arm-III, but there was no statistically significant difference between the arms.

## Discussion

This study documents that the use of microscope for malaria diagnosis significantly reduces prescriptions of antimalarial drugs when compared to use of clinical algorithm alone. The majority of health workers in Arm-I adhered to the blood smear results provided to them. In contrast, the practice of giving antimalarial drugs to patients with negative blood smears was significantly more frequent in other studies [18-22]. One possible reason for the added trust in the laboratory results in this study maybe the involvement of the prescribers in the microscopy training together with laboratory staff.

Prescription of antibiotics was also influenced by microscopy as patients in Arm-I with negative blood smears were more likely to be prescribed antibiotics compared to patients with positive blood smears. This is consistent with findings from northeast Tanzania [18]. Restricting antimalarial drugs to patients with positive blood smears obviously enhances clinicians to consider antibiotics treatment of potential bacterial diseases e.g. pneumonia another important cause of infant and childhood mortality.

The findings from this study, further confirm the limited usefulness of clinical algorithms alone in malaria diagnosis as has been shown earlier [11,12,23,24]. However the algorithms seemed to improve the ability of health workers to consider other alternative diagnosis, resulting in an increase in frequency of antibiotic prescriptions in the clinical diagnosis intervention arm compared to the control arm. This is in agreement with findings from Kenya [12] and Uganda [25] where between 30% to 50% of children were classified and treated according to IMCI as having both pneumonia and malaria.

The current malaria treatment guidelines for Tanzania recommend any under-five child with fever or history of fever within the last 24 hours without evidence of other diseases should be treated for malaria even with a negative blood smear/RDT for malaria parasites [26]. The rationale for this policy is that in areas of very high malaria transmission, malaria is the most likely cause of their illness and withholding antimalarial drugs in this highly vulnerable group will lead to poor outcomes. In this study, the reduced prescription rate of antimalarial prescription in Arm-I compared to arms II and III did not result in any negative effect parasitologically or clinically. Patients in Arm-I were not more likely to re-attend to the health facilities between day 1–6 and even fewer patients had parasitaemia on day 7 compared to the other two arms. A reason for this observation may be better adherence to treatment in patients with malaria when they know that a parasitological test has confirmed the diagnosis. Similar results from Uganda showed that withholding antimalarial treatment in febrile children with negative blood smears was safe and saved 1,600 antimalarial treatments in 601 children over an 18-month period [27].

There was sub-optimal quality of antimalarial drug prescriptions among PHC health workers characterized by incorrect doses, incorrect duration and high rate of amodiaquine use in some health facilities. These prescribing patterns are similar to the results of previous cross sectional studies in Tanzania [4,6]. These characteristics of inappropriate drug use cause particular concern for the development of resistance. Therefore, interventions to improve rational use of antimalarial drugs in a sustainable and cost-effective way are of crucial importance[28].

Clustering on PHC facilities was accounted for in the analysis and the relatively small number of PHC facilities randomized to each arm might have reduced the power of the study to show some important differences between study arms. However, this was partly compensated for by including a large number of patients. Selecting PHC facilities that were geographically accessible from Dar es Salaam might have introduced a selection bias. However the selected facilities were not different from those not included in the study in terms of number and quality of staff and availability of drugs.

The overall accuracy of blood smear reading in this study was relatively low with a large variation in the estimated positive predictive values and negative predictive values in different PHC facilities. False positive smears were frequently reported, indicating a tendency among laboratory workers to report a blood smear as positive when they are in doubt. Also false negative smears were reported in some patients with high parasite counts, maybe due to insufficient reading time and poor smear quality. One such child developed severe malaria. These reading inaccuracies will result in delayed malaria diagnosis, severe illness and also undermine the management of non-malaria associated illness. A national laboratory training programme in Ghana demonstrated improvement in diagnostic accuracy of microscopy in PHC facilities [15]. The partly different outcome to this study may have different reasons such as relatively short period of training and differences in qualifications of the laboratory technicians in the two studies as well as increased work load to clinical staff in PHC facilities which had no laboratory staff.

## Conclusion

The findings of the present study indicate that microscopy supported malaria diagnosis reduces over-prescription of antimalarial drugs. However, the accuracy of the microscopy readings in this study was suboptimal despite the extra training and supervision provided by the study personnel. Lack of qualified laboratory personnel at PHC facilities in Tanzania and the relatively short training period of the staff may have contributed to the shortcomings. This calls for a simple malaria diagnostic tool for PHC facilities and whether the role of RDTs may be more efficient than microscopy in such peripheral health care settings needs to be investigated. Clearly laboratory tests confirming the presence of malaria parasites will be increasingly required, especially in the context of possible future reduction in the incidence of malaria related disease [29,30]. This will influence the risks of not treating patients with false negative smear readings and should be weighed against benefits from restricted prescription of antimalarial drugs, i.e. reduced drug costs and drug pressure and improved management of alternative fever etiologies.

## Competing interests

The authors declare that they have no competing interests.

## Authors' contributions

BN planned the study, conducted the field trial, data analyses and interpretation, and wrote the report. MM conducted the field trial and data analysis. MW contributed to the study planning and design, interpretation of results and write up of the manuscript. MGP assisted in study planning, designed data analyses and interpretation. AM participated in study planning and coordinated the field trial. ZP participated in study planning and coordinated the field trial. LLG involved in study planning and review of manuscript. GT involved in study planning and review of manuscript. AB contributed to study planning, design, and coordination, interpretation of results and preparation of the manuscript. All authors read and approved the final manuscript.

## Financial support

The study received financial support from the SIDA/Sarec bilateral project between KI and MUCHS(Bil-Tz 16/98 75007059)
